# Impact of correction of ischemic mitral valve on left ventricular reverse remodeling

**DOI:** 10.1186/1749-8090-8-S1-O280

**Published:** 2013-09-11

**Authors:** A Nikolic, M Mirocevic

**Affiliations:** 1 Department of Cardiac Surgery, Clinical Center of Montenegro, Podgorica, Montenegro

## Background

Chronic ischemic mitral regurgitation (IMR) is frequent complication of myocardial infarction. It is associated with high cardiovascular morbidity and mortality. We examined the effects of surgery of ischemic mitral valve on reverse remodeling of left ventricle, impact on functional state of patients after operation, assessment of early morbidity and mortality.

## Methods

Coronary artery bypass grafting (CABG) and surgical correction of ischemic mitral valve were performed in 52 patients with coronary artery disease (CAD) and IMR and 52 patients with CAD and IMR underwent only CABG. In both groups they had moderate or severe mitral insufficiency. Serial transthoracic and transesophageal echocardiograms were performed before surgery, after 6 months and after 12 months to assess grade of MR, left ventricular and left atrial remodeling, left ventricle ejection fraction. Clinical follow up - NYHA functional classification, survival, events were assessed after 1-year follow up. Statistical test used were Mann Whitney U test, student’s t-test, c2 test.

## Results

LV end-systolic dimension decreased from 49.0 to 41.3 mm in combined operation group and from 45.4 to 42.0 mm in only CABG patients at one year follow up. LV end-diastolic dimension decreased from 65.1 to 59.3 mm in patients with both CABG and mitral valve surgery and from 60.6 to 58.1 mm in patients with only CABG after 12 months. Reverse remodeling of left ventricle occurred more frequently in group of patients with combined surgery, 71.8% vs 41.5% in only CABG group. Ejection fraction increased for 7.5% in combined group and 2.5% in only CABG group. NYHA class improved in both groups of patients, but with more significant result in combined operation group.

## Conclusion

In patients with ischemic mitral valve better results of left ventricular reverse remodeling were obtained with combined CABG and mitral valve surgery than in patients without surgical correction of ischemic mitral valve.

